# Grade of recurrent *in situ* and invasive carcinoma following treatment of pure ductal carcinoma *in situ* of the breast

**DOI:** 10.1038/sj.bjc.6601704

**Published:** 2004-03-30

**Authors:** R R Millis, S E Pinder, K Ryder, R Howitt, S R Lakhani

**Affiliations:** 1 Hedley Atkins Cancer Research UK Breast Pathology Laboratory, Guy's Hospital, London SE1 9RT, UK; 2 Department of Histopathology, University of Nottingham, Nottingham City Hospital, Hucknal Road, Nottingham NG5 1PB, UK; 3 Academic Oncology Unit, Guy's Hospital, London SE1 9RT, UK; 4 Department of Cellular Pathology, Southampton General Hospital, Southampton SO16 6YD, UK; 5 The Breakthrough Toby Robins Breast Cancer Research Centre, ICR and the Royal Marsden Hospital, London SW3 6JB, UK

**Keywords:** breast carcinoma, ductal carcinoma *in situ* (DCIS), histological grade

## Abstract

The grade of recurrent *in situ* and invasive carcinoma occurring after treatment of pure ductal carcinoma *in situ* (DCIS) has been compared with the grade of the original DCIS in 122 patients from four different centres (The Royal Marsden Hospitals, London and Sutton, 57 patients; Guy's Hospital, London, 19 patients; Nottingham City Hospital, 31 patients and The Royal Liverpool Hospital, 15 patients). The recurrent carcinoma was pure DCIS in 70 women (57%) and in 52 women (43%) invasive carcinoma was present, which was associated with an *in situ* element in 43. In all, 19 patients developed a second recurrence (pure DCIS in 11 and invasive with or without an *in situ* element in eight). The majority of invasive carcinomas followed high-grade DCIS. There was strong agreement between the grade of the original DCIS and that of the recurrent DCIS (*κ*=0.679), which was the same in 95 of 113 patients (84%). The grade of the original DCIS showed only fair agreement with the grade of recurrent invasive carcinoma (*κ*=0.241), although agreement was stronger with the pleomorphism score of the recurrent carcinoma (*κ*=0.396). There was moderate agreement, in recurrent invasive lesions, between the grade of the DCIS and that of the associated invasive element (*κ*=0.515). Other features that showed moderate or strong agreement between the original and recurrent DCIS were necrosis and periductal inflammation. The similarity between the histological findings of the original and subsequent DCIS is consistent with the concept that recurrent lesions represent regrowth of residual carcinoma. In addition, although agreement between the grade of the original DCIS and that of any subsequent invasive carcinoma was only fair, there is no suggestion that low-grade DCIS lesions progress to higher grade lesions or to the development of higher grade invasive carcinoma. This is in agreement with immunohistochemical and molecular data indicating that low-grade and high-grade mammary carcinomas are quite different lesions.

The best treatment for ductal carcinoma *in situ* (DCIS) of the breast has yet to be determined, although there is an increasing body of evidence that radiotherapy is of benefit after wide local excision (Julien *et al*, 2000; UK Coordinating Committee on Cancer Research (UKCCCR) Ductal Carcinoma *in situ* Working Party, 2003). The increase in the number of cases diagnosed since the introduction of mammographic screening has emphasised the considerable diversity exhibited by these lesions. Several histopathological classifications have been proposed during the past two decades (Lagios *et al*, 1989; Holland *et al*, 1994; Poller *et al*, 1994; Silverstein *et al*, 1995, 1996; Scott *et al*, 1997; Sloane *et al*, 1998). These are all based primarily on nuclear morphology and also, in some, on the presence or absence of necrosis or cell polarisation. The histological type, together with the lesion size, and, perhaps of most significance, the size of the margin of clearance have been found to be the best determinants of the risk of recurrence (Lagios *et al*, 1989; Poller and Ellis, 1995; Silverstein *et al*, 1996, 1999; Bijker *et al*, 2001a; Douglas-Jones *et al*, 2002). Approximately half of the recurrent lesions remain *in situ*, but half show evidence of invasion. Owing to the potential for metastasis, the latter are of considerable significance as the chance of successful eradication of the disease may have been lost. In several studies the incidence of recurrence, at least in the short term, has been found to be higher following high-grade (poorly differentiated) DCIS than following low-grade (well differentiated) lesions (Lagios *et al*, 1989; Schwartz *et al*, 1992; Bellamy *et al*, 1993; Solin *et al*, 1993; Sneige *et al*, 1995; Silverstein *et al*, 1996; Fisher *et al*, 1999). Moreover, in some studies, the incidence of an invasive recurrence has been found to be higher after high-grade DCIS than after low-grade lesions (Lagios *et al*, 1989; Bellamy *et al*, 1993). This suggests that more radical initial treatment may be indicated for high-grade DCIS than for low-grade DCIS.

Histopathological studies of invasive carcinoma with an associated *in situ* component have shown a close link between the grade of the *in situ* and that of the invasive component (Lampejo *et al*, 1994; Goldstein and Murphy, 1996; Gupta *et al*, 1997). Furthermore, the clinical outcome is related to the histology of the DCIS. Those with a well-differentiated *in situ* component associated with their invasive carcinoma have a more favourable prognosis than patients with poorly differentiated lesions (Lampejo *et al*, 1994; Gupta *et al*, 1997). Studies of recurrent *in situ* and invasive carcinoma following treatment for pure *in situ* carcinoma have shown a high degree of correlation between the grades of the original and recurrent tumours (Barnes *et al*, 2000; Bijker *et al*, 2001b). If recurrent lesions following pure DCIS are always of the same grade, it can be argued that low-grade DCIS is not only less likely to recur and to develop invasive disease but also that, if it does recur, the chance of successful treatment is still there. If this is so, such lesions could be managed in a less radical manner.

The present study was undertaken to try to establish the nature of recurrent lesions, both *in situ* and invasive, occurring following the treatment of pure DCIS.

## MATERIALS AND METHODS

The files of patients treated for DCIS were reviewed and those in whom a recurrent carcinoma had occurred after an interval of at least 6 months from initial treatment were selected. The recurrent lesions were within the ipsilateral residual breast or, in a few cases, within the mastectomy scar. Only cases in which the slides of both the primary and recurrent tumour were available for review were included in the study. The 122 patients studied were seen and treated at the Royal Marsden Hospital, London and Surrey (RMH) (57 patients; initial diagnosis between 1975 and 1990), Guy's Hospital, London (Guy's) (19 patients; initial diagnosis 1975–1994), Nottingham City Hospital (NCH) (31 patients; initial diagnosis 1975–1996) and the Royal Liverpool Hospital (RLH) (15 patients; initial diagnosis 1982–1996). Preliminary findings relating to some of the NCH patients have been included in previous presentations (Barnes *et al*, 2000).

The age of the patients ranged from 24 to 81 years (Guy's patients, 24–81 years, median 54 years; RMH patients, 28–72 years, median 52 years; NCH patients, 29–75 years, median 52 years; RLH patients, 37–64 years, median 54 years). Treatment in the majority of cases was surgical excision either alone or in combination with adjuvant tamoxifen or radiotherapy or both. In a minority, initial treatment was simple or radical mastectomy. The time to first recurrence ranged from 6 to 177 months (Guy's patients, 18–177 months, median 69 months; RMH patients, 7–125 months, median 39 months; NCH patients, 6 months–143 months, median 56 months; RLH patients, 12–180 months, median 36 months).

The following features were recorded in the primary *in situ* tumours: nuclear grade, architectural pattern, necrosis and periductal fibrosis and inflammatory (lymphoplasmacytic) reaction. In the recurrent lesions, the nuclear grade of the *in situ* carcinoma and, in most cases, also the presence of necrosis, the architectural pattern and periductal changes were noted, as was the grade of the invasive lesion where present. The slides were reviewed separately by two of the authors in all cases, and where there was disagreement they were then studied together or in some cases a third author was consulted.

The nuclear grade of the *in situ* lesions was based on the features used in the United Kingdom National Health Service Breast Screening Programme (UK NHSBSP) and the European Commission Working Group on Breast Screening Pathology (Sloane *et al*, 1998). High-grade DCIS is composed of cells with pleomorphic nuclei, which are usually large, show marked variation in size and shape, have coarse chromatin and prominent nucleoli and are irregularly spaced. Mitoses are usually frequent and both individual cell necrosis and phagocytosis may be present. Low-grade DCIS consists of cells with monomorphic nuclei that are usually small, spherical and central. Nucleoli are inconspicuous and the chromatin is uniform. Mitoses are rare, as is individual cell necrosis. Intermediate grade DCIS is composed of cells that have intermediate features and cannot readily be assigned to either of the above categories. There is mild to moderate pleomorphism that is less than in high-grade lesions, but the monotony of low-grade DCIS is absent and nucleoli are often present. In lesions where differing ducts showed variation in nuclear grade, all grades present were recorded. Architectural pattern was described as solid, cribriform, micropapillary or intracystic papillary. When more than one pattern was present, each was recorded. Necrosis was scored as 0 absent, 1 mild, 2 moderate or 3 marked (usually of comedo type). Periductal fibrosis and inflammatory reaction were both scored as 1 absent/mild, 2 moderate or 3 marked. The grade of recurrent invasive carcinoma was based on the Nottingham modification of Bloom and Richardson grading (Elston and Ellis, 1991).

## RESULTS

The grade of the DCIS in the original lesions is shown in Table 1

**Table 1:** Comparison of original and recurrent DCIS grades

	**Recurrent DCIS grade (highest present)^a^**			
**Original DCIS grade (highest present)**	**1**	**2**	**3**	**Total**
1	6	1	1	8
2	2	16	2	20
3	1	11	73	85
Total	9	28	76	113

a Only first recurrence included. *χ*^2^=97.76, *P*<0.001, *κ*=0.679.

. Over 75% of cases were high grade and only eight cases (6.5%) of pure low-grade DCIS were present in the study.

At first recurrence, 70 (57%) cases recurred as pure DCIS and 52 (43%) contained invasive carcinoma. Of those with invasion, 43 recurred as invasive with an associated *in situ* component and nine as invasive without an *in situ* component identified. One of the invasive lesions with an *in situ* component was micro-invasive (<1 mm in maximum dimension) and is not included in any further analysis of invasive tumours. As shown in Table 1, in the 113 cases in which DCIS was present in the first recurrence, when the highest DCIS grade of both the first and recurrent lesion were considered, there was close agreement between the grades (*κ*=0.679). The grades were the same in 95 of 113 cases (84%). The first lesion contained more than one grade of DCIS in 13 cases and in seven cases there was more than one grade in the second DCIS (see Table 2

**Table 2:** Numbers of cases with mixed DCIS grade

	**Number of cases with mixed DCIS grades**			
	**3+2+1**	**3+2**	**3+1**	**2+1**
Original DCIS	1	7	1	4
Recurrent DCIS	1	5	0	1

). If all grades were considered, there were nine cases in which the grade of the second DCIS was not seen in the first lesion. In seven, the grade was lower and in two it was higher.

In all, 19 patients had a second recurrence. In nine of these patients, both recurrences were pure *in situ*, in four both contained *in situ* and invasive disease, in four the first was pure *in situ* but the second contained invasive carcinoma and in two patients, although invasion was present in the first recurrence, it could not be identified in the second. Again, there was close agreement between the grade of the DCIS in the original lesion and the second recurrence (data not shown).

The majority of the invasive recurrences followed high-grade DCIS (42 of 55; 76%) and only four occurred in patients in whom the original lesion was pure low-grade DCIS. There was only fair agreement between the highest grade of the original DCIS and the grade of the first detected recurrent invasive tumour, whether this was in the first or subsequent recurrence (*κ*=0.241). As shown in Table 3

**Table 3:** Comparison of original DCIS grade with recurrent invasive grade

	**Recurrent invasive grade^a^**			
**Original DCIS grade (highest)**	**1**	**2**	**3**	**Total**
1	2	1	1	4
2	3	5	1	9
3	1	21	20	42
Total	6	27	22	55

a First detected invasive tumour whether in first or second recurrence. *χ*^2^=15.82, *P*=0.003, *κ*=0.241.

, in 27 of the 55 cases (49%) the grades were the same, in the rest the invasive grade was lower in 25 and higher in three. In 21 of the former the DCIS was grade 3, while the invasive carcinoma was grade 2. Table 4

**Table 4:** Comparison of grades of DCIS and invasive carcinoma in recurrent lesions

	**Associated recurrent invasive grade^a^**			
**Recurrent DCIS grade (highest present)**	**1**	**2**	**3**	**Total**
1	3	1	0	4
2	3	8	1	12
3	0	11	19	30
Total	6	20	20	46

a First detected invasive tumour whether in first or second recurrence. *χ*^2^=26.99, *P*<0.001, *κ*=0.573.

shows that the DCIS grade of the recurrent *in situ* carcinoma associated with the invasive carcinoma was related to the grade of the invasive carcinoma (*κ*=0.513). The grade was the same in 30 of 46 cases (65%). The invasive grade was higher in two patients and lower in 14. In 11 of the latter the DCIS was grade 3 and the invasive carcinoma was grade 2.

The different components of the grade of the invasive carcinoma (tubule formation, nuclear pleomorphism and mitoses) were analysed separately in relation to the grade of both the original and the recurrent *in situ* carcinomas. Fair agreement was found between the original DCIS grade and the pleomorphism score (*κ*=0.396) (Table 5

**Table 5:** Comparison of original DCIS grade with pleomorphism score of recurrent invasive cancer

	**Recurrent invasive grade pleomorphism score^a^**			
**Original DCIS grade (highest present)**	**1**	**2**	**3**	**Total**
1	1	1	2	4
2	0	5	4	9
3	0	6	36	42
Total	1	12	42	55

a First detected invasive carcinoma whether in first or second recurrence. *χ*^2^=21.12, *P*<0.001, *κ*=0.396.

), but not with either tubule formation or the mitotic score. The recurrent DCIS was related to all the three components, but most strongly to pleomorphism (*κ*=0.485) (Table 6

**Table 6:** Comparison of recurrent DCIS grade with pleomorphism score of recurrent invasive cancer

	**Associated recurrent invasive carcinoma pleomorphism score^a^**			
**Recurrent DCIS grade (highest)**	**1**	**2**	**3**	**Total**
1	1	3	0	4
2	0	6	7	13
3	0	3	26	29
Total	1	12	33	46

a First detected invasive carcinoma whether in first or second recurrence. *χ*^2^=23.68, *P*<0.001, *κ*=0.485.

).

As shown in Tables 7

**Table 7:** Comparison of the presence of necrosis in original and recurrent DCIS

	**Necrosis recurrent DCIS**				
**Necrosis original DCIS**	**0**	**1**	**2**	**3**	**Total**
0	15	6	5	4	30
1	8	4	3	4	19
2	1	2	6	5	14
3	4	2	2	16	24
Total	28	14	16	29	87

*χ*^2^=28.04, *P*<0.001, *κ*=0.380.

and 8

**Table 8:** Comparison of the degree of inflammation in original and recurrent DCIS

	**Inflammation recurrent DCIS**			
**Inflammation original DCIS**	**1**	**2**	**3**	**Total**
1	33	15	1	49
2	16	9	1	26
3	0	6	6	12
Total	49	30	8	87

*χ*^2^=34.26, *P*<0.001, *κ*=0.340.

, the scores for necrosis and inflammatory reaction within the original and recurrent DCIS were also associated (*κ*=0.380 and 0.340, respectively). No such association was found for architectural pattern or periductal fibrosis. The number of cases with a mixed pattern made analysis of the former difficult.

In addition (data not shown), high-grade DCIS was associated with high scores for necrosis, inflammatory reaction and fibrosis, and also with the presence of a solid growth pattern, either alone, or in combination with other patterns. High scores for necrosis, periductal fibrosis and inflammation and the presence of a solid growth pattern, in the original DCIS, were also associated with high-grade recurrent DCIS and with the presence of invasion in the recurrence.

## DISCUSSION

This study of tumours from 122 patients who developed relapse of DCIS or invasive progression shows strong agreement between the grade of initial DCIS and subsequent recurrent *in situ* lesions (*κ*=0.679). In 84% of cases they were the same. There is also an association, although less strong, between the grade of initial DCIS and the grade of subsequent invasive recurrences (*κ*=0.241). These findings add strength to the report of similar findings in a subsection (*n*=31) of the cases included in the current study (Barnes *et al*, 2000). They also support the findings of the recent EORTC study on recurrent lesions from 116 patients (Bijker *et al*, 2001b). In the latter study, the DCIS was found to be the same in the 70% of initial and recurrent lesions, and in 53% of cases the grade of the recurrent invasive carcinoma was the same.

In our study, where the grade of the initial DCIS and that of either the recurrent DCIS or the recurrent invasive tumour were not the same, the grade of the subsequent tumour was more often lower than higher. In a significant number of cases the initial DCIS grade was 3, whereas the recurrent invasive tumour was grade 2. A similar association was found with the recurrent DCIS associated with the invasive carcinoma. This association of grade 3 DCIS with grade 2 invasive carcinoma has been noted previously (Lampejo *et al*, 1994; Barnes *et al*, 2000). Of more importance was the finding in one case each of recurrent invasive carcinoma grade 2 and 3 following an initial low-grade DCIS lesion. Conversely, however, invasive grade 1 carcinomas followed three cases of intermediate grade DCIS and one of high-grade DCIS.

The criteria used for grading DCIS and invasive carcinomas are not the same. The grading system for DCIS used in this study was that used in the UK NHSBSP, which is based only on nuclear morphology (Sloane *et al*, 1998). The additional evaluation of tubule formation and number of mitoses in the grading of invasive carcinomas may well account for some of the discrepancies between DCIS grade and that of the invasive tumours. Indeed, a closer association was found between the nuclear pleomorphism score for the recurrent invasive carcinoma and the original DCIS grade (*κ*=0.396 and 0.485, respectively).

A possible reason for the lack of agreement between the original and recurrent lesions is that the second lesion is a new primary tumour rather than a recurrence of the previous one. One way to try to evaluate this is to see whether or not the lesions arose at the same site. It was difficult in many cases to ascertain the site of recurrent lesions in the breast. Where this information could be found, it was in the same quadrant in the majority of cases (>80%). In the EORTC study, 11% of recurrences occurred in a different quadrant from the original lesion, but this was not found to affect the level of agreement (Bijker *et al*, 2001b).

The EORTC study also evaluated the immunohistochemical profile of the initial and subsequent DCIS and found 63% agreement with staining for oestrogen and progesterone receptors, p53 and HER2/neu oncoprotein (Bijker *et al*, 2001b). Another study has looked at the chromosomal alterations of *in situ* carcinomas and recurrent lesions using comparative genome hybridisation (CGH). A high level of agreement in 18 of 20 cases was found, although the mean number of CGH changes was higher in the recurrent than the initial lesions (Waldman *et al*, 2000). Both of these studies add strength to the theory that the recurrent lesions following excision of DCIS are closely related to the original disease and almost certainly represent re-growth of residual tumour. This emphasises the importance of clear margins at the time of initial surgery.

The cases for this study were selected on the basis that the DCIS had recurred and inevitably included a large proportion of high-grade DCIS and only eight low-grade lesions. Most previous studies of recurrence following treatment of DCIS have found a higher incidence of both DCIS and invasive carcinoma following high-grade DCIS, at least in the short term (Lagios *et al*, 1989; Schwartz *et al*, 1992; Bellamy *et al*, 1993; Solin *et al*, 1993; Sneige *et al*, 1995; Silverstein *et al*, 1996; Fisher *et al*, 1999). In the EORTC study, although the risk of DCIS recurrence was found to be significantly related to the initial histology, the risk of invasive recurrence was not (Bijker *et al*, 2001a). However, patients with poorly differentiated DCIS had a significantly higher risk of developing distant metastases and of death, which must reflect the higher grade of invasive carcinomas associated with this type of DCIS. Furthermore, within well-differentiated DCIS, those with a clinging or micropapillary pattern had significantly lower rates of recurrence than those with a cribriform pattern.

The findings in the current study that high-grade DCIS is associated with high scores for periductal necrosis, fibrosis and inflammatory reaction and with the presence of a solid growth pattern is to be expected. An association between the scores for these features in the original DCIS and with the occurrence of high-grade DCIS or the presence of invasion in recurrent lesions is also to be anticipated.

The number of cases of recurrent invasive carcinoma in this study is small, particularly following low-grade DCIS. As recurrence following low-grade DCIS may occur long after diagnosis of the original lesion, long-term follow-up may be necessary to detect such lesions (Page *et al*, 1995; Solin *et al*, 1995). Although this study shows only fair agreement between the grade of the original DCIS and that of the subsequent invasive tumour, there is no suggestion in this or the EORTC study that low-grade lesions often progress to a higher grade. This is in agreement with the consistency in malignancy grade, which has been found in studies of primary invasive mammary carcinoma when compared to both local and distant recurrences (Hitchcock *et al*, 1989; Millis *et al*, 1998).

There is growing evidence that low-grade and high-grade carcinomas are two distinctly different entities. Immunohistochemical studies of a range of markers show that they have different profiles. Both *in situ* and invasive low-grade lesions express oestrogen and progesterone receptors, but are usually negative for oncoproteins (HER/neu and p53). High-grade lesions exhibit the reverse (Hitchcock *et al*, 1989; Gullick *et al*, 1991; Barnes *et al*, 1993; Millis *et al*, 1996; Ellis *et al*, 1999). Molecular genetic studies find that low-grade lesions often demonstrate 16q deletions, while high-grade lesions have allelic losses of more chromosomal areas frequently including 1p, 1q, 6q, 9p, 11p, 13q and 17q. Intermediate grade DCIS tends to show combinations of the patterns associated with low- and high-grade lesions (Buerger *et al*, 1999a and 1999b; Ellis *et al*, 1999; Roylance *et al*, 2002).

This study adds weight to the idea that recurrence following treatment of DCIS represents re-growth of residual disease. It also supports the concept that the grade of mammary carcinoma does not progress with time. Further studies with longer follow-up and, if possible, greater numbers of low-grade tumours are needed finally to answer the question. Owing to the natural history of low-grade DCIS, it may be necessary to combine the results from many centres in order to obtain sufficient numbers of recurrent lesions for analysis.
